# Theoretical Perspectives on Balance Training and the Gut–Muscle–Brain Axis in Aging

**DOI:** 10.3390/brainsci16040432

**Published:** 2026-04-21

**Authors:** Ahmad Zandi, Johannes Burtscher, Peter Federolf

**Affiliations:** 1 Department of Sport Science, University of Innsbruck, 6020 Innsbruck, Austria; ahmad.zandi@student.uibk.ac.at; 2 Department of Psychiatry, Psychotherapy, Psychosomatics and Medical Psychology, University Clinic for Psychiatry II, 6020 Innsbruck, Austria; johannes.burtscher@i-med.ac.at

**Keywords:** balance training, neuromuscular adaptation, postural control, aging, sarcopenia, gut–muscle–brain axis, microbiota and inflammation

## Abstract

With growing global life expectancy, age-related physical problems, including balance impairments, are becoming more prevalent, increasing the risk of falls, mobility limitations, and loss of independence. This review summarizes current evidence on how balance may be influenced and improved by training modalities including reactive, strength-based, and functional exercises, through neuromuscular adaptations relevant to postural control and functional stability in older adults. Emerging evidence suggests that gut microbiota may influence neuromuscular health via neuroimmune, metabolic, and mitochondrial pathways across the gut–muscle–brain axis. However, most studies focus on muscle metabolism, inflammation, and systemic physiological processes rather than direct assessments of balance or postural control. Gut dysbiosis has been associated with sarcopenia and impaired physical function, although evidence linking microbiota alterations to balance outcomes remains limited and mainly observational. Exercise has beneficial effects on neuromuscular function and gut microbial composition, including increased diversity and metabolite production. While exercise-induced neuromuscular adaptations are well supported experimentally, little direct evidence shows the contribution of gut-related mechanisms to balance regulation. Overall, neuromuscular and gut-related processes seem to be associated with balance capacity in older adults; however, further mechanistic and interventional studies are required to clarify the role of the gut–muscle–brain axis for balance.

## 1. Introduction

With life-expectancies increasing worldwide, fall-related injuries among older adults have emerged as a major global health challenge. Falls are the leading cause of injury-related morbidity and mortality among older adults and, according to the WHO Global Report on Falls Prevention in Older Age, 28–35% of individuals aged 65 years and older experience at least one fall annually. This proportion rises to 32–42% among those aged ≥70 [1]. Such falls frequently result in serious outcomes—fractures, head trauma, hospitalization, long-term disability, and loss of functional independence [1]. In the United States alone, more than 36 million falls are reported annually among adults aged 65 and older, resulting in over 32,000 deaths and generating healthcare costs exceeding $50 billion each year [2]. This public health and economic burden is expected to intensify as people live longer. Identifying modifiable risk factors is thus critical for designing effective fall prevention strategies tailored to the needs of older adults [3]. Balance training has been shown to improve postural control and is associated with reduced fall risk in older adults, likely mediated by neuromuscular and functional adaptations [4,5]. These adaptations are supported by experimental and clinical evidence demonstrating that balance training significantly improves multiple components of balance performance, including static, dynamic, proactive, and reactive balance in older adults [5]. High-quality evidence from randomized controlled trials indicates that structured exercise interventions significantly reduce both the rate of falls and the risk of falling in older adults [4]. However, the underlying mechanisms remain only partially understood. The interplay between different organ systems may contribute to these improvements, although the relative contribution of each system remains unclear.

The current article reviews available evidence on the gut–muscle–brain axis and discusses potential links to balance and postural stability in older adults. Current evidence linking the gut microbiota to functional outcomes is largely derived from studies focusing on muscle mass, inflammation, and related metabolic processes rather than direct assessments of balance or postural control [6,7]. Importantly, no studies to date have directly assessed both gut microbiota composition and balance or postural control outcomes in humans. Therefore, the gut–muscle–brain axis should currently be considered merely a conceptual framework integrating findings from multiple disciplines. This review aims to address this gap by synthesizing evidence across neuromuscular and microbiome domains, while clearly distinguishing between well-established neuromuscular mechanisms and emerging pieces of evidence related to gut and microbiome contributions.

### Literature Search Strategy

Electronic databases including PubMed, Web of Science, and Google Scholar were searched for relevant studies published between 2000 and 2025. The search strategy combined free-text keywords and, where applicable, Medical Subject Headings (MeSH), including “balance training”, “postural control”, “aging”, “sarcopenia”, “gut microbiota”, “neuromuscular adaptation”, and “neuroinflammation.” Priority was given to peer-reviewed human studies, systematic reviews, and meta-analyses addressing (i) balance and postural control, (ii) neuromuscular adaptations to exercise, and (iii) microbiome-related mechanisms relevant to muscle function and aging. In addition, forward and backward snowballing were employed, and the literature collections of the authors were interrogated. We aimed to include representative and widely cited studies across disciplines, with study selection based on relevance to the predefined thematic scope, as well as methodological quality.

## 2. Age-Related Decline in Balance and Postural Stability

### 2.1. Conceptual Definitions: Balance, Aging, and Sarcopenia

Balance is a multifaceted motor skill that plays a crucial role in maintaining functional independence, especially at older age. It reflects the capacity to maintain bodily stability during both static and dynamic tasks through the coordinated interplay of sensory systems (visual, vestibular, and somatosensory inputs), central processing, and appropriate neuromuscular responses [8,9]. From a biomechanical standpoint, balance represents a state of postural equilibrium in which destabilizing forces are effectively countered. Clinically, impaired balance is strongly associated with an increased risk of falls, making its assessment and enhancement essential in geriatric healthcare [10]. Such impairments have also been widely reported as indicators of functional decline in older adults. This integrative perspective highlights the coordinated interaction of sensory and motor systems underlying postural control. While these mechanisms are well established, balance control in aging is increasingly considered a complex phenomenon involving interactions between neuromuscular and sensory systems rather than isolated deficits [11].

Aging is a complex biological process characterized by the progressive decline in physiological integrity, which is associated with impaired function and increased vulnerability. It is associated with the accumulation of molecular and cellular damage over time. Aging involves multifaceted changes at the genetic, cellular, and systemic levels, including telomere shortening, oxidative stress, and dysregulation of metabolic pathways, which collectively contribute to the gradual loss of homeostasis and resilience [12,13]. These systemic alterations affect not only muscle function but also immune/inflammatory and metabolic pathways associated with whole-body functional decline, particularly through mechanisms such as chronic low-grade inflammation (“inflammaging”) and age-related physiological dysregulation [14,15]. However, despite the recognized role of multisystem interactions in postural control [11], their specific contribution to balance and postural control remains incompletely understood due to the complex and multifactorial nature of these processes, involving multiple interacting sensory and neural systems.

Sarcopenia is defined as a progressive and generalized skeletal muscle disorder characterized by accelerated loss of muscle mass and function. Nearly all older adults experience some age-related loss of muscle, and 10–40% meet the criteria for sarcopenia as a clinical condition at the age of 65. Sarcopenia is associated with adverse outcomes including physical disability, poor quality of life, and mortality. A consensus definition and diagnostic criteria were revised in 2019 by Cruz-Jentoft and colleagues to improve clinical recognition and management of sarcopenia [16]. In addition to its musculoskeletal consequences, sarcopenia has been associated with chronic low-grade inflammation and alterations in gut microbiota composition [6,7]. These associations suggest potential links between microbial, metabolic, and muscular processes; however, current evidence is primarily derived from studies focusing on muscle mass, inflammation, and metabolic regulation rather than direct assessments of balance or postural control [6,7]. Accordingly, the relevance of microbiota-related mechanisms for balance regulation should currently be considered indirect. The available evidence is largely associative and influenced by multiple confounding factors, including diet, physical activity, and comorbidities. Therefore, causal relationships remain to be established through well-controlled interventional studies, particularly randomized controlled trials (RCTs) [6,17].

### 2.2. Physiological Background: Aging and Balance

As individuals age, balance control undergoes multifactorial decline, which has traditionally been thought to be primarily due to progressive deterioration in the integration of sensory, motor, and cognitive systems. Age-related impairments in proprioception, vestibular function, and visual acuity—each fundamental for postural control—have been well documented [9]. Muscle strength and power commonly decline in older adults, particularly in the lower limbs, leading to delayed postural responses and difficulties in compensating for perturbations [18,19]. Furthermore, structural and functional impairments in the neuromuscular system, such as decreased muscle spindle sensitivity and slower transmission in afferent pathways, further compromise timely balance corrections [18]. Age-associated neural degeneration not only impairs central processing speed and coordination, but also diminishes the capacity for anticipatory and reactive adjustments necessary for maintaining stability [20]. In addition, cognitive decline, comprising reduced attention and executive function, compromises balance—especially in dual-task situations—highlighting the interdependence between cognition and postural control [21]. Taken together, these findings indicate that age-related balance impairment cannot be attributed to a single physiological system but rather reflects the involvement of multiple interacting physiological systems, requiring an integrative perspective for accurate interpretation and intervention design [11]. This interplay becomes particularly evident when older adults are required to divide attention between a cognitive task and a balance challenge, which often results in impaired performance in one or both domains [21].

Growing fall risk is multifactorial, encompassing intrinsic factors such as muscle weakness, balance impairment, sensory deficits, and cognitive decline, as well as extrinsic environmental hazards [3]. Age-related physiological and neurological declines substantially increase the risk of falls among older adults, underscoring the urgency of effective preventive strategies in geriatric care. Gait and balance disorders represent one of the primary contributing factors to fall risk in later life, as age-related deterioration in multisensory integration, muscle strength, and neuromotor response capacity compromises postural stability and mobility [8]. This decline highlights the need for precise balance assessment and targeted intervention strategies to mitigate fall risk in older people. Moreover, musculoskeletal disorders such as osteoarthritis have been shown to impair balance stability, particularly during stair ascent or descent [22]. In individuals with sarcopenia, the risk of falls is further increased, for example, over threefold in the ilSIRENTE cohort [23], even after adjusting for age, comorbidities, and physical activity level. Collectively, the interaction between sensorimotor and musculoskeletal decline intensifies the vulnerability during everyday ambulation and facilitates falls with advancing age. Interventions aiming to preserve or restore neuromuscular function through targeted exercise programs effectively enhance balance performance and reduce fall risk in older adults, as shown by meta-analytic evidence demonstrating significant improvements in balance function and reductions in falls following exercise interventions [24]. Beyond these well-established neuromuscular and sensorimotor mechanisms, the potential contribution of systemic biological processes to age-related functional decline has recently begun to be explored, including inflammatory and metabolic pathways. However, current evidence linking these systemic factors—particularly gut microbiota-related mechanisms—to balance and postural control remains largely indirect and inferential, as most studies focus on associations with muscle function, frailty, and inflammation rather than direct assessments of balance outcomes [6,14,17]. These observations provide a physiological basis for exploring how systemic factors may interact with neuromuscular mechanisms involved in balance regulation in older adults. Postural control is a complex process that depends on the integration of multiple sensory inputs and motor responses across interacting physiological systems [11].

### 2.3. The Gut–Muscle–Brain Axis: Regulating Balance and Vulnerability to Sarcopenia?

Recent investigations highlight the potential influence of the gut–muscle axis on neuromuscular performance and balance regulation in older people, suggesting that this concept should be extended to a broader gut–muscle–brain framework. Alterations in gut microbiota diversity and function have been observed in older adults with sarcopenia, with notable shifts in specific microbial taxa and associated metabolic pathways [25,26]. These microbial changes have been associated with increased systemic inflammation and impaired amino acid metabolism, both of which may contribute to muscle degradation and frailty [25]. Age-related gut dysbiosis has been linked to elevated pro-inflammatory cytokines, such as interleukin-6 (IL-6) and tumor necrosis factor-alpha (TNF-α), which can interfere with muscle protein synthesis and mitochondrial function [26,27]. While these mechanisms are well supported in the context of muscle metabolism and systemic inflammation, their direct implications for neuromuscular coordination and balance control remain insufficiently understood and require further investigation [6,7]. It is biologically plausible that these inflammatory mediators may influence neuromuscular function; however, direct evidence linking these processes to anticipatory postural adjustments or measurable balance outcomes in humans remains limited [18].

In contrast, gut-derived short-chain fatty acids (SCFAs), particularly butyrate, have been suggested to exert anti-inflammatory effects and may support muscle health and insulin sensitivity, thereby contributing to systemic conditions that are potentially favorable for postural balance [26,27]. Furthermore, neuroimaging studies have reported associations between gut microbiota composition and activity in sensorimotor and interoceptive brain regions [28], indicating a possible interaction between microbial metabolites and the central nervous system. However, these findings do not establish direct evidence of improved postural control, and their functional relevance to balance regulation remains to be clarified. Although SCFAs may influence central nervous system functions, including neuroimmune responses and neurotransmitter regulation, direct evidence linking these effects to balance performance is still sparse and largely indirect [29,30]. Emerging evidence suggests that gut-related factors may influence physiological processes such as inflammation and metabolic regulation, as well as skeletal muscle function, although their role in postural control needs to be better investigated [6,25].

Physical exercise, particularly resistance and balance training, has been shown to be associated with beneficial changes in gut microbiota composition, including increases in microbial diversity and SCFA production [27]. However, these findings should be interpreted with caution, as the available evidence is largely derived from observational or pilot studies [31]. Exercise is also associated with reductions in circulating pro-inflammatory markers, such as IL-6, in older adults [32,33]. Beyond well-established neuromuscular and sensorimotor mechanisms, emerging evidence suggests that systemic biological processes, including inflammaging—a state characterized by persistent immune activation and metabolic dysregulation—and gut microbiota-related pathways, may contribute to age-related functional decline [14]. In parallel, alterations in gut microbiota composition have been associated with frailty, reduced physical performance, and overall health status in older adults [17]. However, these associations are primarily correlational, and current evidence remains insufficient to establish causal relationships. The proposed gut–muscle axis suggests that microbiota-related mechanisms may influence muscle mass, strength, and physical performance; however, this evidence is influenced by multiple confounding factors, with limited direct investigation in human populations [6,18]. This connection is therefore primarily inferred from neuromuscular and physiological research rather than directly demonstrated in microbiome-focused studies. Changes in gut microbiota may contribute to neuromuscular decline and sarcopenia in aging adults [6,27]. Notably, a recent randomized controlled trial reported preliminary evidence suggesting potential improvements in postural balance following oral butyrate supplementation in older adults [34]. However, these findings remain preliminary, and the underlying mechanisms—particularly those related to gut–muscle and potentially gut–muscle–brain interactions—require further elucidation [6,7].

## 3. Postural Stability Mechanisms and the Gut–Muscle–Brain Axis in Aging

### 3.1. Sensorimotor, Neuromuscular, and Emerging Contributors to Postural Decline

Postural stability requires continuous integration of multisensory information and the execution of coordinated neuromuscular responses to maintain upright orientation during both static and dynamic conditions. Effective postural control relies on the proper functioning of the visual, vestibular, and proprioceptive systems, as well as the ability of the central nervous system to interpret sensory inputs and generate timely corrective actions [11,35]. With aging, the physiological systems underlying postural control undergo progressive decline. Reductions in sensory accuracy, slower neuromuscular activation, impaired central processing, and decreased muscle strength and power collectively compromise both anticipatory and reactive postural adjustments. At the peripheral level, aging is associated with reduced muscle spindle sensitivity, slower peripheral nerve conduction, and diminished reflex modulation efficiency, leading to less precise sensorimotor integration and delayed corrective responses [36].

At the central level, older adults exhibit a reduced capacity to dynamically reweight visual, vestibular, and proprioceptive inputs in response to changing environmental demands, thereby limiting postural adaptability [35]. Age-related reductions in motor-unit firing rates further impair the rapid generation of stabilizing forces required to maintain balance [37]. In addition, lower-limb muscles—particularly at the ankle and hip—demonstrate reduced force production capacity, which limits the effectiveness of corrective strategies during perturbations [38]. Older adults also show delayed activation of compensatory muscles and increased antagonist coactivation. While such coactivation may transiently increase joint stiffness, it reduces movement efficiency and slows reactive adjustments that are critical for maintaining stability [39,40]. Collectively, these neuromuscular changes disrupt both anticipatory and reactive postural adjustments, increasing instability and fall risk during daily activities [10].

Aging is also associated with measurable declines in peripheral sensory function, including proprioceptive acuity and vestibular sensitivity, which contribute to altered postural performance. Experimental evidence indicates that age-related declines in sensory and motor function—particularly in proprioceptive acuity—are associated with increased postural sway and altered balance performance, especially under conditions requiring complex sensory integration [41]. In addition, reductions in proprioceptive sensitivity and impaired integration of sensory inputs have been linked to less accurate control of upright stance [42]. Declines in vestibular and proprioceptive function further reduce the reliability of sensory information required for postural adjustments, although balance training may partially mitigate some of these deficits [41]. Importantly, sensorimotor and neuromuscular mechanisms remain the most consistently supported determinants of postural stability, as evidenced by their strong and reproducible associations with balance performance across experimental and clinical studies. These findings further reinforce the central role of sensorimotor mechanisms in postural stability in older adults [11].

Recent literature suggests that additional biological mechanisms, including alterations in gut function and microbiota composition, may be associated with age-related changes in motor function [6,43]. These mechanisms may influence postural stability indirectly by modulating inflammation, neuromuscular function, and metabolic processes relevant to balance control, although current evidence is primarily derived from studies on muscle function and systemic physiology rather than direct assessments of balance [6,25]. In conclusion, a direct causal relationship between gut microbiota and postural control has not yet been established. Therefore, while the integration of gut-related factors into models of postural control represents a conceptually promising direction, these mechanisms should currently be interpreted as complementary rather than primary contributors when compared to well-established sensorimotor and neuromuscular determinants of balance impairment [11].

### 3.2. Inflammatory and Metabolic Disruptions from Gut Dysbiosis

A growing body of observational and experimental literature suggests that age-related gut dysbiosis is associated with metabolic and inflammatory pathways relevant to skeletal muscle physiology; however, its role in postural control remains largely indirect and not yet fully established [6,14,25]. Importantly, direct evidence linking gut microbiota to postural control or balance performance in humans is currently lacking.

Mechanistic, preclinical, and experimental evidence indicates that alterations in gut microbiota composition may influence host metabolic and inflammatory processes. However, the extent to which these pathways translate into functional outcomes related to motor control or balance remains unclear and requires further investigation.

(i) Reduced microbial diversity and SCFA production: Aging has been associated with reduced gut microbial diversity and altered microbial composition, particularly in individuals with reduced muscle mass. Lower levels of SCFAs, especially butyrate, have been reported and linked to muscle mass and function in older adults [6,25,44]. While these findings support a role of the gut microbiota in skeletal muscle physiology, their direct relationship with postural control has not been demonstrated.
(ii) Metabolic endotoxemia and inflammation: Age-related alterations in gut barrier integrity may contribute to increased translocation of microbial-derived components and the development of chronic low-grade systemic inflammation [14]. This inflammatory state has been associated with alterations in metabolic regulation and muscle-related processes [6]. However, direct evidence linking these inflammatory processes to balance impairments or postural instability in humans is limited.
(iii) SCFA-mediated metabolic and mitochondrial regulation: SCFAs, particularly butyrate, have been shown to play a role in regulating metabolic processes, including mitochondrial function and cellular energy homeostasis, which are important for maintaining skeletal muscle health [6,25,44]. Nevertheless, the translation of these molecular effects into measurable improvements in postural control or balance performance remains uncertain since it has not been directly demonstrated.
(iv) Neurophysiological and gut–brain interactions: Gut-derived metabolites may influence central and peripheral physiological processes involved in motor function and sensorimotor integration, as suggested by the broader concept of the gut–brain axis [44]. However, evidence directly linking these pathways to postural control outcomes in older adults.
(v) Sarcopenia-associated microbiota profiles: Distinct alterations in gut microbiota composition have been observed in sarcopenic individuals, including reduced microbial diversity and changes in the abundance of beneficial bacterial taxa [6,25,44]. These findings suggest an association between gut microbiota and muscle-related outcomes such as strength and physical performance, although, their role in balance regulation remains hypothetical.

Collectively, the available evidence—largely based on associative human studies and preclinical models, with limited well-controlled interventional data—suggests that gut dysbiosis may be linked to metabolic and neuromuscular processes relevant to skeletal muscle function; however, these interactions remain poorly understood. Direct experimental evidence linking gut microbiota alterations to postural control or balance outcomes in humans is still lacking. Therefore, while the gut–muscle–brain axis is considered a promising conceptual framework, its specific role in postural control and age-related balance impairments has yet to be fully elucidated, given the complex integration of sensory, motor, and cognitive systems in maintaining stability [11]. Future well-controlled human studies combining microbiome profiling with objective measures of postural stability are required to clarify these relationships.

### 3.3. Gut–Muscle–Brain Axis: Mechanisms and Conceptual Framework

The concept of the gut–muscle–brain axis has been proposed as a communication network linking the gastrointestinal microbiota, skeletal muscle and the central nervous system. This framework suggests that physiological communication within this axis is mediated through interconnected neural, endocrine, immune, and metabolic pathways, contributing to the regulation of systemic homeostasis [45,46]. The gut microbiota is considered a key contributor to this system through the production of a wide range of bioactive metabolites. Among these, SCFAs, including butyrate and propionate, are produced via microbial fermentation of non-digestible dietary substrates. These metabolites have been associated with regulatory roles in host metabolism, immune modulation, and energy homeostasis, and may serve as important signaling molecules linking gut microbial activity to systemic and neuromuscular processes [47]. Communication between the gut microbiota and the central nervous system is thought to occur via multiple pathways, including neural (e.g., vagal pathways), immune, and endocrine signaling. Microbiota-derived metabolites and related compounds may influence central nervous system function, including neuroplasticity and behavioral regulation. In this context, alterations in microbiota composition, often referred to as dysbiosis, have been associated with both gastrointestinal and psychological disturbances, suggesting a potential role of the gut microbiota in brain–gut interactions [48].

Chronic low-grade inflammatory has been proposed as one of the mechanisms linking age-related changes in the gut microbiota with systemic physiological decline. This process is characterized by an imbalance between pro- and anti-inflammatory responses and may be driven, in part, by the accumulation of endogenous molecules derived from cellular damage and reduced efficiency of clearance mechanisms such as autophagy and mitophagy. Over time, this state has been suggested to contribute to the development and progression of chronic diseases [49]. In addition, metabolic endotoxemia, defined as increased circulating levels of lipopolysaccharide (LPS), has been proposed as a mechanistic link between the gut microbiota and metabolic disorders. Preclinical evidence suggests that elevated LPS levels may promote systemic inflammation and contribute to the development of insulin resistance, obesity, and related metabolic disturbances, potentially via modulation of the innate immune system [50]. The gut–muscle axis has also been proposed as an important component of this network, particularly in the context of aging and sarcopenia. Emerging evidence suggests that alterations in gut microbiota composition may be associated with changes in muscle mass, strength, and physical performance. Preclinical and clinical studies, including fecal microbiota transplantation, indicate that modulation of the gut microbiota can influence host muscle phenotype, supporting a potential causal relationship; however, the extent to which these findings translate to human physiology remains under investigation [43].

Mechanistically, the interaction between the gut microbiota and skeletal muscle is thought to involve multiple pathways, including regulation of inflammatory signaling, energy metabolism, mitochondrial function, and neuromuscular integrity. These pathways provide a plausible biological basis for the influence of gut-derived signals on muscle health and physical performance, although further mechanistic studies are required to fully elucidate these interactions. Skeletal muscle itself functions as an endocrine organ by secreting a variety of signaling molecules known as myokines. These include interleukin-6 (IL-6), brain-derived neurotrophic factor (BDNF), and irisin, which have been implicated in the regulation of systemic metabolism, lipid oxidation, and inflammatory processes. The secretion of these myokines is largely dependent on muscle contraction, indicating that physical activity may play a central role in modulating inter-organ communication within this axis [51]. Physical exercise has been proposed as a key non-pharmacological factor influencing the gut–muscle–brain axis. Available evidence suggests that aerobic exercise may increase gut microbiota diversity and modulate the relative abundance of specific bacterial taxa, including members of the Firmicutes phylum. These exercise-induced changes in microbiota composition may represent one of the potential pathways through which exercise exerts beneficial effects on host metabolism and central nervous system function [48].

**Figure 1:**
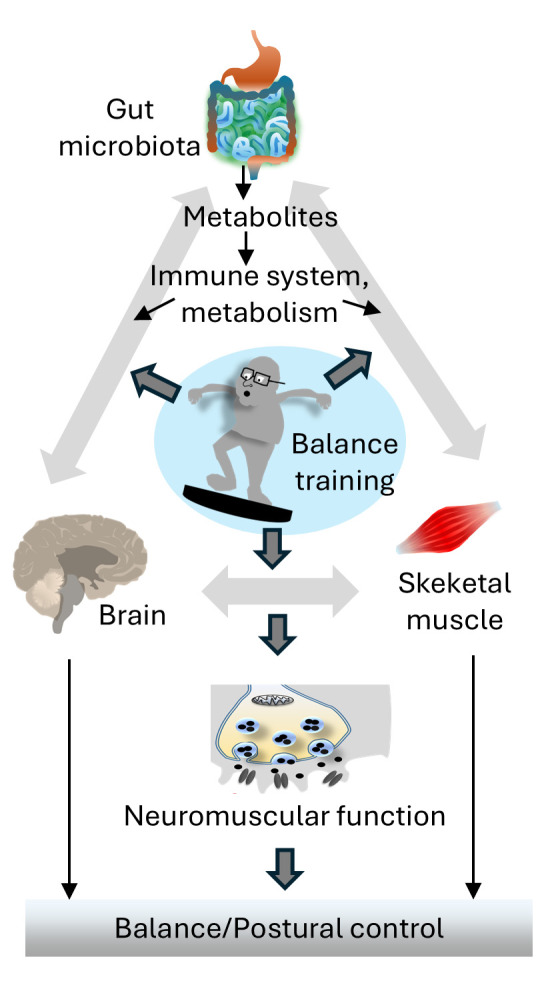
Conceptual illustration of the gut–muscle–brain axis highlighting neural, immune, and metabolic pathways linking gut microbiota to skeletal muscle and central nervous system function. The figure emphasizes the role of exercise as a key modulatory factor influencing microbiota composition, myokine signaling, and neuroendocrine regulation, ultimately contributing to neuromuscular function and balance control in aging.

Furthermore, exercise has been associated with the modulation of several neuroendocrine peptides, including neuropeptide Y, peptide YY, ghrelin, and leptin, which are involved in the regulation of appetite, energy balance, and gastrointestinal function. These findings suggest that exercise may contribute to the regulation of systemic homeostasis through integrated metabolic and signaling pathways [52]. In addition, physical activity may help attenuate some of the adverse effects associated with obesity-related dysbiosis and chronic low-grade inflammation. Obesity has been associated with alterations in gut microbiota composition and increased inflammatory tone, whereas exercise appears to promote a shift toward a more favorable microbial profile and improved metabolic regulation, although the exact mechanisms remain to be fully elucidated [52]. Overall, the available evidence suggests that the gut microbiota may represent one of several contributing factors within a complex network linking the gut, brain, and skeletal muscle. This system integrates metabolic, immune, and neural signals to regulate physiological function, and its dysregulation has been associated with aging, sarcopenia, and functional decline. Targeting the gut microbiota through lifestyle interventions such as physical exercise, as well as dietary strategies and microbial-based therapies, may therefore represent a promising approach for supporting neuromuscular function and promoting healthy aging. These mechanisms may provide an indirect biological basis linking gut–muscle–brain interactions to postural control and balance performance, as conceptually illustrated in Figure 1. Importantly, no studies to date have directly assessed both microbiota composition and balance outcomes in humans, and the proposed links remain largely conceptual.

## 4. Effects of Balance Training

### 4.1. Neuromuscular and Neural Adaptations to Balance Training in Older Adults

Balance training interventions have generally been shown to improve postural control and may contribute to reductions in fall risk in older adults, although the magnitude and consistency of these effects vary across studies. Perturbation-based balance training (PBT), such as treadmill-based or unstable-surface paradigms, enhances task-specific balance abilities and reduces fall risk in fall-prone older adults [53,54]. Strength and power training enhances lower-limb muscle strength and power, contributing to improved stability during both static and dynamic tasks [55]. Tai Chi training improves balance performance in both single- and dual-task conditions and enhances cognitive-motor integration, with Yang-style Tai Chi showing the greatest benefits in older adults [56,57]. Dual-task training programs, combining motor and cognitive challenges, improve older adults’ ability to maintain balance under concurrent cognitive and motor demands and are associated with improvements in postural stability and reductions in fall risk in some intervention studies [58,59].

In general, well-designed training interventions have been reported to enhance lower-limb force generation and the rate of force development, enabling faster and more effective corrective actions during destabilizing events [39,60]. Exercise-based interventions, particularly those involving repeated perturbations, can induce neuromuscular adaptations associated with changes in muscle activation patterns and the reorganization of muscle synergies during balance-related tasks. Repeated exposure to perturbations has been shown to refine motor strategies and improve coordination of muscle groups involved in postural control [61]. In addition, balance training can promote central neural adaptations, such as structural plasticity in cortical regions associated with visual and vestibular processing, which are known to contribute to postural orientation and stability [62]. These neuromuscular adaptations may interact with broader systemic physiological processes, including metabolic and inflammatory pathways, although the extent to which such interactions influence balance outcomes remains unclear. However, evidence regarding the extent to which such training consistently improves clinical balance outcomes remains mixed, and the underlying neuromuscular mechanisms are only partially understood. Furthermore, while these neuromuscular and neural adaptations are supported by experimental evidence, their translation into consistent functional improvements across diverse older populations is debated. The potential contribution of microbiome-mediated metabolic or inflammatory pathways to balance training outcomes remains speculative and is supported mainly by indirect, associative, or preclinical evidence.

### 4.2. Balance Training and the Gut–Muscle–Brain Axis

Emerging evidence suggests that the effectiveness of balance and resistance training may be influenced not only by neuromuscular adaptations but also indirectly and context- dependently by the composition, diversity, and metabolic activity of the gut microbiota. Conversely, a healthy gut microbiome may potentially support physiological processes relevant to muscle metabolism, protein synthesis, mitochondrial function, and may contribute to reducing systemic inflammation, thereby potentially contributing to functional outcomes. However, direct evidence linking these effects to measurable improvements in postural balance in older adults remains limited.

The gut–muscle axis—a bidirectional signaling network—has been proposed as a potential regulator of muscle homeostasis, whereby physical activity can directly stimulate muscle function and also favorably influence gut microbiota composition and metabolic output, including the production of microbial metabolites such as SCFAs [6,7]. Thereby, it may potentially contribute to supporting muscle mass, strength, and functional performance in both aging and clinical populations [43,63]. In addition, the gut microbiome may influence neuromuscular junction integrity and CNS function, including cognition and executive functions [64,65]. Consequently, the gut–muscle–brain axis can be considered a hypothetical biological framework for understanding training and rehabilitation outcomes.

**Figure 2:**
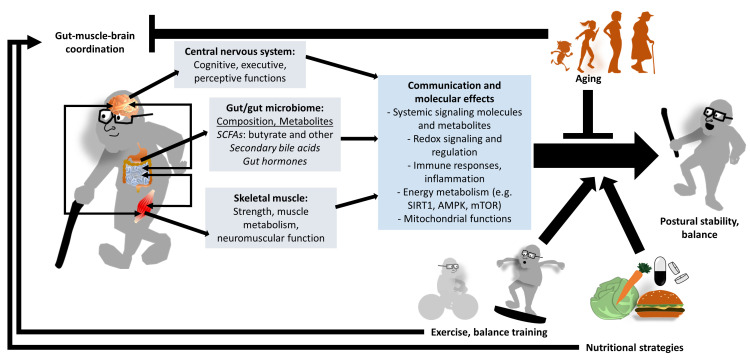
Conceptual representation of the gut–muscle–brain axis modulation by age, exercise/balance training, and nutrition. Gut microbiota generate metabolites, including SCFAs, which influence systemic signaling pathways, mitochondrial activity in distant tissues, muscle metabolism, and neuromuscular and central nervous system functions. Exercise (particularly balance-focused) and nutrition strategies beneficial to the microbiome may modulate this axis and could potentially contribute to postural stability, although this framework remains largely conceptual and not fully empirically validated.

Various mechanisms involved in this interaction (Figure 2) have been extensively discussed in the literature:

(i) Exercise-induced microbiome modulation: Aerobic and resistance exercise have been reported to increase microbial diversity and may enhance populations of beneficial SCFA–producing taxa [31,66]. The resulting elevation in SCFA production may potentially support mitochondrial functions, reduce inflammation, and be associated with anabolic signaling in skeletal muscle [67]. However, the direct causal effect of these microbiome changes on postural control or balance metrics in humans has not yet been firmly established.
(ii) Microbiome-dependent variability in training adaptations: Oral butyrate supplementation (300 mg/day for 16 weeks) has been reported to improve postural balance, handgrip strength, and gait speed, while reducing circulating markers of intestinal permeability in older adults [34]. While these findings are promising, they are based on a controlled intervention with a relatively limited sample, and it remains unclear to what extent the observed improvements are mediated directly through SCFA-related neuromuscular effects versus indirect metabolic or muscular adaptations.
(iii) SCFA-mediated molecular pathways: Short-chain fatty acids, particularly butyrate, have been suggested to modulate muscle and mitochondrial metabolism through pathways involving AKT/mTOR and AMPK signaling. Experimental studies suggest that SCFA supplementation may activate AKT/mTOR/S6K1 and AMPK/PGC-1α pathways and may promote improvements in muscle mass and mitochondrial biogenesis [68]. In addition, review evidence supports that SCFAs regulate muscle protein metabolism, mitochondrial function, and inflammatory pathways through mechanisms including AMPK and mTOR signaling [69]. However, these findings are primarily derived from preclinical studies, and their direct relevance to postural control in humans remains to be established.

These pathways may support mitochondrial biogenesis, ATP production, and overall metabolic function, although current evidence is largely derived from preclinical studies [68,70]. Importantly, most of the available preclinical evidence linking microbiome-related pathways to muscle and metabolic outcomes still needs to be confirmed in humans. Nevertheless, combining balance or resistance training with microbiota-targeted nutritional strategies (e.g., prebiotics, probiotics, high-fiber diets, or targeted SCFA supplementation) may hold promise for synergistic effects on muscle performance and, potentially, postural stability.

Clinical evidence is provided by the PROMOTe trial [71], which demonstrated that prebiotic supplementation, when paired with exercise and increased amino acid intake, resulted in microbiome modulation (e.g., increased Bifidobacterium abundance). However, no significant improvements in physical function were observed, although cognitive outcomes improved. These findings highlight the potential of integrating exercise with microbiome modulation, while underscoring that robust evidence for improvements in postural control remains limited and preliminary.

## 5. Discussion

### 5.1. Key Insights

Balance impairments in older adults arise from a convergence of sensory decline, neuromuscular deterioration, and systemic biological disturbances—not solely from musculoskeletal factors. Classical explanations centered on weakened muscles, slower reflexes, and reduced sensory accuracy remain valid, but should be considered in the context of multiple biological alterations associated with aging. An important aspect discussed in this review is that alterations in the gut microbiome may indirectly influence physiological processes relevant to neuromuscular function. Age-related reductions in microbial diversity, loss of SCFA-producing taxa, and increases in pro-inflammatory microbial profiles have been associated with changes in muscle metabolism, systemic inflammation, and neuromuscular function [27,72,73]. These alterations may be linked to mechanisms that are relevant to balance regulation—such as motor-unit responsiveness, mitochondrial energy metabolism, and sensorimotor integration—direct evidence linking these microbiome-related changes to postural control outcomes in humans lacking.

Current evidence also suggests that exercise and the gut microbiome influence each other. Physical activity, including aerobic and resistance exercise, has been proposed to modulate gut microbiota composition and metabolic activity, while gut-derived metabolites such as SCFAs have been shown in experimental models to support muscle metabolism and mitochondrial function [31,68,70]. Importantly, skeletal muscle activation remains the primary driver of exercise-induced adaptations, whereas microbiome-related responses are more likely to represent secondary or complementary processes rather than primary mechanisms.

Taken together, these findings support the view that balance impairment in aging is a multifactorial phenomenon shaped by the interaction of sensory, neuromuscula, metabolic, and inflammatory processes. milieus rooted in gut ecology and reinforcing the role of the gut–muscle–brain axis. Microbiome-related mechanisms in the context of balance function need to be elucidated. Future studies integrating microbiome profiling with objective assessments of postural control and well-controlled exercise interventions could clarify these relationships and their potential clinical relevance.

### 5.2. Gaps in Current Research

Despite increasing evidence linking gut health, systemic inflammation, and neuromuscular function, direct evidence connecting gut–muscle–brain interactions to balance capacity remains limited. Balance control is a complex process involving the integration of multiple sensory, motor, and cognitive systems [11]. However, the extent to which these interacting systems are systematically investigated in balance-focused interventions is still insufficient.

(i) Lack of Mechanistic Integration: Many balance-focused studies primarily report improvements in functional outcomes, while providing limited insight into the underlying neurophysiological mechanisms. Although some studies have utilized electromyography to assess neuromuscular responses, comprehensive investigations of central mechanisms—including neural network adaptations and higher-level motor control processes—remain limited. As a result, the mechanisms underlying training-induced improvements in postural control are not yet fully elucidated [11].
(ii) Limited Integration of Systemic and Microbiome-Related Mechanisms: Emerging evidence suggests that gut microbiome composition and its metabolites may be associated with physiological pathways involved in muscle metabolism, systemic inflammation, and neuromuscular function. Age-related alterations in microbial diversity, including reductions in SCFA-producing taxa, have been linked to processes relevant to muscle function and host metabolism. However, these systemic and microbiome-related factors are rarely incorporated into balance research frameworks, limiting a more comprehensive mechanistic understanding of balance impairments in older adults.
(iii) Dissociated Microbiome and Exercise Science: Although exercise interventions have been reported to influence gut microbiome composition, and microbiome alterations have been associated with muscle-related outcomes, these research domains are usually investigated separately. This separation limits the ability to explore potential interactions between exercise-induced adaptations and microbiome-related processes. Integrating these perspectives may be important to improve mechanistic understanding and to support the development of more comprehensive intervention strategies targeting balance and mobility in older adults.
(iv) Longitudinal, multimodal studies integrating microbiome profiling with structured balance or resistance training interventions remain scarce. Studies, such as the DEMGUTS pilot randomized controlled trial protocol [31], aim to investigate the effects of different exercise modalities on gut microbiome composition and gut-derived metabolites in older adults. However, empirical findings are not yet available, and the extent to which such approaches can clarify mechanistic links between microbiome alterations and functional outcomes remains to be determined.

### 5.3. Clinical Relevance

The evidence synthesized in this review supports a potential conceptual shift toward biologically integrated balance rehabilitation frameworks in older adults. Traditional training modalities such as perturbation-based exercises, strength and power training, and sensorimotor conditioning remain essential and are strongly supported by neuromechanical research. However, their effectiveness may be influenced by underlying biological states, including systemic inflammation, metabolic status, and gut microbiome composition. From a practical perspective, this highlights the potential value of combining established balance training programs with adjunctive strategies, including nutritional interventions such as prebiotics, probiotics, synbiotics, and high-fiber dietary approaches. Such combined approaches have been proposed as potential complementary strategies to support neuromuscular function, reduce inflammation, and enhance metabolic health, although their effectiveness in improving postural stability in humans remains to be fully established. A key limitation in the current literature is that no studies have directly assessed both gut microbiota composition and balance or postural control outcomes in human populations.

These considerations may be particularly relevant in fall prevention programs for older adults, where individualized interventions could be tailored based on physiological, metabolic, and possibly microbiome-related factors. Integrating such approaches into clinical practice may help optimize rehabilitation outcomes and address inter-individual variability in training responsiveness. Furthermore, incorporating biological markers, such as inflammatory indicators, metabolic profiles, and microbiome composition, may assist in identifying individuals who are less responsive to conventional interventions and may benefit from alternative or adjunctive strategies. However, these applications remain largely theoretical and require validation through well-designed experimental and longitudinal studies.

## Data Availability

No new data were created or analyzed in this study. Data sharing is not applicable to this article.
